# Neurosteroids: a lifelong impact on brain health

**DOI:** 10.3389/fnbeh.2025.1644615

**Published:** 2025-09-30

**Authors:** Najah L. Walton, Jamie L. Maguire

**Affiliations:** Department of Neuroscience, Tufts University School of Medicine, Boston, MA, United States

**Keywords:** neurosteroids, allopregnanolone, development, aging, exercise

## Abstract

Neurosteroids are critical regulators of brain function, exerting profound effects on neurodevelopment, emotional regulation, cognition, and resilience to stress across the lifespan. Synthesized endogenously in the brain and peripheral tissues, neurosteroids modulate neural circuits through both genomic and non-genomic mechanisms. This review synthesizes current evidence on the roles of neurosteroids from fetal development through advanced biological aging, emphasizing their involvement in neuronal plasticity, synaptic modulation, myelination, and neurogenesis. We explore how neurosteroid dysregulation contributes to mood and cognitive disorders and highlight age- and sex-related changes in neurosteroid synthesis which may impact risk. Lifestyle factors including diet, exercise, and mindfulness are also examined for their ability to modulate neurosteroidogenesis and promote brain health. By integrating findings across developmental stages and physiological states, we underscore the functional roles of neurosteroids as modulators of emotional and cognitive states across the lifespan, and advocate for deeper investigation into neurosteroid-based intervention for across indications and throughout the lifespan.

## Introduction

Across the lifespan the brain undergoes dynamic structural and functional transformations, driven by the complex interplay between innate neurobiological processes and extrinsic environmental factors (Fandakova et al., 2025; Hoagey et al., 2025). Among the key modulators of these processes are neurosteroids, a class of endogenous steroids synthesized in the brain and peripheral tissues, that exert profound effects on neural function through both genomic and non-genomic mechanisms (Balthazart et al., 2018; Colciago et al., 2020; Rebas et al., 2017). Neurosteroids have been shown to exert a wide range of effects and have robust therapeutic potential (Zorumski et al., 2025). Neurosteroids, such as allopregnanolone, pregnenolone, progesterone, and dehydroepiandrosterone (DHEA), play essential roles in supporting neuronal development, stress regulation, and emotional wellbeing throughout the lifespan, which will be highlighted throughout this review.

While neurosteroids are fundamental to maintaining central nervous system (CNS) homeostasis (Lloyd-Evans and Waller-Evans, 2020), they are also highly sensitive to a variety of external influences, including diet, physical activity, psychological stress, environmental toxins, and pharmacological interventions (Antonoudiou et al., 2022; Locci et al., 2017; Sonnenblick et al., 2018). These factors can significantly modulate neurosteroid synthesis and signaling, leading to diverse outcomes that range from neuroprotection to increased vulnerability to neurodevelopmental disorders, reproductive dysfunction, and psychiatric illnesses such as anxiety, depression, and post-traumatic stress disorder (PTSD) (Evans-Strong et al., 2024; Vacher et al., 2025; Walton et al., 2023). Furthermore, alterations in neurosteroid levels and neurosteroidogenesis are increasingly recognized as biomarkers of affective states, aging, and cognitive decline, underscoring their importance in both health and disease.

The aim of this review is to synthesize current clinical and preclinical evidence describing the multifaceted roles of neurosteroids across the lifespan. We examine their involvement in early brain development, placental and fetal physiology, adolescent brain maturation, reproductive transitions such as puberty and menopause, and age-related decline in neurosteroid synthesis in both humans and preclinical models. Additionally, we explore how lifestyle factors and interventions including diet, exercise, and meditation, modulate neurosteroid levels and influence brain health. By integrating findings across developmental stages and physiological contexts, this review highlights the critical importance of neurosteroids as regulators of brain function and potential therapeutic targets for enhancing neurological and psychiatric outcomes. Deepening our understanding of how these forces influence one another will provide opportunities to develop impactful solutions.

## Major neurosteroid classes

Neurosteroids are endogenously synthesized by neurons and glial cells de novo from cholesterol or metabolized from steroid hormone precursors (testosterone, progesterone, or corticosterone) (Lloyd-Evans and Waller-Evans, 2020; Mellon and Griffin, 2002; Mellon and Vaudry, 2001). Endogenous production of neurosteroids is governed by a series of enzymatic reactions. Neurosteroids can be synthesized de novo from cholesterol, whereby cholesterol is transported into steroidogenic mitochondria by a translocator protein (TSPO) and subsequently cleaved by Cytochrome P450 side-chain cleavage enzymes (P450SCC). This cleavage results in the formation of pregnenolone, that gets exported from mitochondria and catalytically converted by the enzyme 3β-Hydroxysteroid dehydrogenase (3β-HSD) to progesterone. Key rate limiting enzymes, 5α-reductases, are responsible for converting progesterone to 5α-dihydroprogesterone. 3α-Hydroxysteroid dehydrogenase is then responsible for the bidirectional reaction in which Allopregnanolone is produced. Pregnenolone may also be reduced by P450c17 enzymes into 17α-hydroxyprogesterone. These same enzymes are involved in converting 17α-hydroxyprogesterone into dehydroepiandrosterone (DHEA). Hydroxysteroid sulfotransferase (HST) and Steroid Sulfatase (STS) mediate the production of the sulfated form of DHEA, dehydroepiandrosterone sulfate (DHEAS), and its reduction to DHEA (Mellon and Griffin, 2002; Mellon and Vaudry, 2001).

Neurosteroids can be classified by either structure or function (Table 1). Structural classifications include the division of neurosteroids into one of three groups: (1) pregnane, (2) androstane, and (3) sulfated (Legesse et al., 2023). Functional classifications are dictated by the inhibitory or excitatory effects of neurosteroids on their target receptors: whereby sulfated neurosteroids are excitatory and both pregnane and androstane neurosteroids exert inhibitory effects (Legesse et al., 2023). These classifications highlight the biological diversity of neurosteroids as intra- and inter-cellular signaling molecules acting as neurohormones and neuromodulators (Zorumski et al., 2025). Neurosteroids serve as ligands to exert non-genomic actions upon plasma membrane receptors (e.g., GABA_*A*_ or NMDA receptors) or genomic actions through the binding of their metabolites to intracellular receptors (e.g., progesterone receptors).

**TABLE 1:** List of neurosteroids by structural and functional classes.

Structural class	Neurosteroid name	Abbreviation	Functional class
Pregnane	Allopregnanolone	ALLO	Inhibitory
	Pregnenolone	PREG	Inhibitory
	Progesterone	PROG	Inhibitory
	Dihydroprogesterone	DHP	Inhibitory
Androstane	3α-androstanediol	3α-diol	Inhibitory
	Dehydroepiandrosterone	DHEA	Excitatory
Sulfated	Dehydroepiandrosterone Sulfate	DHEAS	Excitatory

List of neurosteroids described in this review and their associated abbreviations broken down by structural and functional classes.

## Neurosteroids modulate GABA receptors

Neurosteroids directly influence neuronal functioning via rapid actions on ion channel function. Nonsulfated neurosteroids function as potent positive allosteric modulators of GABA_*A*_Rs, enhancing both the frequency and duration of channel opening in the presence of GABA (Akk et al., 2005). These neurosteroids access GABA_*A*_Rs primarily through lateral diffusion within the plasma membrane, though direct extracellular binding has also been observed (Akk et al., 2005). At low nanomolar concentrations, neurosteroids potentiate receptor function via a highly conserved site on the α-subunit in a GABA-dependent manner (Hosie et al., 2006; Sugasawa et al., 2020). In contrast, at higher (micromolar) concentrations, neurosteroids can directly activate GABA_*A*_Rs independently of GABA through a distinct binding site located at the α/β subunit interface (Chen et al., 2019; Hosie et al., 2006).

Neurosteroid sensitivity is strongly influenced by the receptor subunit composition and GABA concentration. Extrasynaptic GABA_*A*_Rs containing the δ-subunit exhibit greater sensitivity to neurosteroid modulation compared to synaptic receptors incorporating the γ-subunit (Belelli and Lambert, 2005; Carver and Reddy, 2016; Reddy, 2018). δ-Subunit-containing receptors also contribute to tonic inhibition, a form of persistent inhibitory tone activated by low ambient concentrations of GABA, distinguishing them functionally from γ-subunit-containing receptors, which mediate phasic inhibition (Mody and Pearce, 2004). This neurosteroid-mediated modulation of tonic inhibition stands in contrast to the known effects of benzodiazepines, which target synaptic GABA_*A*_Rs through binding at the α1–3, α5, and γ subunit interface. Notably, receptors containing α4 or α6 subunits are insensitive to benzodiazepine binding (Morlock and Czajkowski, 2011; Richter et al., 2012).

Neurosteroids also exert metabotropic, non-ionotropic, effects on GABA_*A*_Rs, potentially through actions on membrane progesterone receptors (mPRs) (Lemons et al., 2025; Vien et al., 2022). Binding of neurosteroids to mPRs initiates a signaling cascade that enhances the surface expression of α4βδ-containing GABA_*A*_Rs (Abramian et al., 2014; Davies et al., 2017; Parakala et al., 2019). This process involves phosphorylation of the β3 subunit at serine residues 408 and 409, a key regulatory modification that both increases receptor trafficking to the membrane and potentiates GABAergic inhibition (Modgil et al., 2017; Parakala et al., 2019). These metabotropic actions are particularly relevant in brain regions involved in emotional regulation such as the hypothalamus, pituitary gland, forebrain, and corpus callosum, where mPRs are highly expressed (Pang et al., 2013). By promoting inhibitory signaling and enhancing neuronal survival, neurosteroids may contribute to the preservation of neural circuits underlying affective tone and emotional resilience.

## Neurosteroids influence neuroplasticity

Neurosteroids have been shown to play important roles in maintaining healthy neuronal functioning by influencing neuroplasticity. Neuroplasticity involves structural or functional remodeling or neurogenesis to adapt to external and internal conditions. Neurosteroids are known to act within the major pathways regulating neuroplasticity (Schverer et al., 2018). Structural plasticity involves alteration in neuronal morphology such as changes in the number or shape of dendritic spines and the length or complexity of dendrites. Neuronal morphology is typically stabilized by components of the cytoskeleton, mainly by its microtubule components that function much like railroad tracks to provide an avenue for transport of local signaling molecules and longer axonal transport (Conde and Cáceres, 2009). Neurosteroids have been shown to act as receptors for several microtubule associated proteins (MAPs), that direct the formation and stability of microtubules within axons and dendrites. For example, pregnenolone increases structural plasticity through modulation of the microtubule cytoskeleton by binding microtubule-associated protein 2, a MAP found specifically in neurons, while DHEA does so by increasing spine density potentially through interactions with intracellular chaperone proteins that regulate calcium signaling and neurotransmission (i.e., σ1 receptors) (Murakami et al., 2000). These structural changes may underlie the impact of neurosteroids on long-term potentiation (LTP), a crucial cellular mechanism underlying learning and memory. Experimental evidence demonstrates that under conditions of cellular stress, synthesis of allopregnanolone increases and contributes to acute inhibition of LTP (Izumi et al., 2024; Zorumski et al., 2014; Zorumski and Izumi, 2012). Importantly, pharmacological inhibition of neurosteroidogenesis prevents these effects, indicating a causal relationship between neurosteroid production and synaptic modulation (Izumi et al., 2024; Zorumski et al., 2014; Zorumski and Izumi, 2012). These actions are considered to mitigate the detrimental effects that stress can have on learning and memory overall, highlighting the neuroprotective role of neurosteroids like allopregnanolone through preserving the integrity of neural circuits.

In contrast to structural plasticity, functional plasticity shapes the efficacy of synapses either in a positive or negative direction through different receptor classes. Pregnenolone sulfate and DHEA sulfate increase functional plasticity by modulating NMDA receptor trafficking and signaling, respectively (Qian et al., 2024; Whittaker et al., 2008). Similarly, DHEA increases functional plasticity by increasing the efficacy of synaptic signaling (Qian et al., 2024). Neurogenesis has been shown to increase in response to pregnenolone sulfate, DHEA, and allopregnanolone (Mellon, 2007). Both pregnenolone sulfate and DHEA increase neurogenesis by acting upon σ1 receptors while allopregnanolone does so through its actions upon GABA_*A*_ receptors (Maninger et al., 2008).

Progesterone impacts another form of neuronal plasticity known as myelination directly through progesterone receptors under basal conditions and in response to neuronal injury (Schumacher et al., 2012). These effects were also demonstrated to occur through indirect actions by enhancing myelin synthesis, achieved by promoting the transcription of genes for myelin-specific proteins (Schumacher et al., 2012). Along with increasing transcription of genes for myelin-specific proteins, neurosteroidogenic enzymes (cytochrome P450 side-chain cleavage enzyme (P450scc) and 3β-Hydroxysteroid dehydrogenase (3β-HSD)) are also increased during myelination (neurosteroidogenic enzymes discussed in this review are listed in Table 2). It has been shown that the spinal cord can induce local neurosteroidogenesis in response to injury (Schumacher et al., 2012). The ability of neurosteroids to influence myelination may involve neurosteroidogenesis in oligodendrocytes. Oligodendrocytes house the enzymatic machinery for neurosteroidogenesis including 5α-reductases, 3α- and 3β-HSD. Expression of these enzymes is tightly temporally regulated whereby 5α-reductases are highly expressed in mature oligodendrocytes while 3β-HSD are highly expressed in pre-progenitors and precursor oligodendrocytes.

**TABLE 2:** Neurosteroidogenic pathway.

Synthesis pathway	Enzyme name	Gene
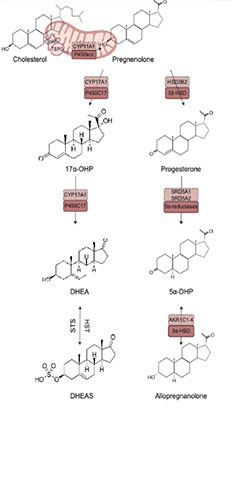	Cytochrome P450 side-chain cleavage enzyme (P450SCC)	CYP11A1
	3β-Hydroxysteroid dehydrogenase (3β-HSD)	HSD3B1
	3α-Hydroxysteroid dehydrogenase (3α-HSD)	AKR1C1, AKR1C2, AKR1C3, AKR1C4
	5α-reductase	SRD5A1, SRD5A2, SRD5A3
	Steroidogenic acute regulatory protein (STAR)	STARD1
	Translocator protein (TSPO)	TSPO
	17β-Hydroxysteroid dehydrogenase III (17β-HSD-3)	HSD17B3

(Left) diagram of the synthesis pathway for the neurosteroids described in this review along with the enzymes required for their synthesis. Corresponding gene names for each neurosteroidogenic enzyme are listed above each enzyme in light salmon.

## Age related fluctuations in neurosteroids

### Fetal development

In utero development has been intimately linked with placental functioning. As an endocrine organ, the placenta not only supports the physical and metabolic needs of the growing fetus but also plays a central role in neurosteroid biosynthesis (Kratimenos and Penn, 2019). These neurosteroids, synthesized and secreted by the placenta, are essential for maintaining pregnancy and regulating the development of the fetal nervous system [for review, see (Kratimenos and Penn, 2019)]. During gestation, neurosteroids such as allopregnanolone exert neuroprotective effects, aiding in the suppression of excess neuronal excitation that could otherwise compromise neural circuit formation (Hirst et al., 2014). Throughout gestation, allopregnanolone levels steadily increase, peaking in the fetal circulation during late pregnancy before declining sharply at birth. This trajectory mirrors maternal neurosteroid levels, which also precipitously decline during parturition. Impaired synthesis of allopregnanolone has been linked to dysregulated central nervous system (CNS) activity in the fetus, increasing the risk of excitotoxicity and long-term neurological deficits (Nicol et al., 2001; Yawno et al., 2007). Impaired neurosteroid signaling in the mother has also been implicated in postpartum depression, which is discussed in more detail below (see Pregnancy and the postpartum period).

Progesterone, another key placental steroid, plays a significant role in promoting brain growth and supporting the proliferation of both neuronal and glial cells in the developing fetal brain (Melcangi et al., 2008; Schumacher et al., 2000, 2012). Additionally, progesterone influences fetal behavior states, including breathing and arousal. Elevated progesterone levels are associated with reduced arousal and diminished electro-ocular activity, while suppression of progesterone results in heightened arousal and increased electro-ocular activity (Crossley et al., 1997; Nicol et al., 1997; Nicol et al., 2001). The third trimester represents a critical window for neurodevelopment, marked by peak levels of pregnenolone, progesterone, and allopregnanolone (Schumacher et al., 2020). This stage is characterized by rapid brain maturation, active myelination, and synaptogenesis, all of which are influenced by the neurosteroid milieu (Clouchoux et al., 2012). Disruption in neurosteroid levels during this period may therefore have profound and lasting effects on neurodevelopmental outcomes.

### Early postnatal development

Postnatal development represents a critical phase in the maturation of synaptic networks, during which changes to neurotransmitter signaling play a fundamental role in shaping neuronal circuitry. Among these, GABAergic signaling undergoes a key developmental transition that is essential for the establishment of functional neuronal networks. In the first postnatal week, GABA acts predominantly as a depolarizing neurotransmitter, exerting profound effects on the developing brain (Ganguly et al., 2001). This excitatory action of GABA facilitates processes such as neuronal migration and maturation (Bortone and Polleux, 2009; Owens and Kriegstein, 2002).

A hallmark of early postnatal development is the shift in GABAergic polarity from depolarizing to hyperpolarizing, occurring within the first two postnatal weeks (Peerboom and Wierenga, 2021). This transformation supports the synchronization and functional integration of emerging neuronal networks (Owens and Kriegstein, 2002). Importantly, this polarity shift coincides with a transient increase in the neurosteroid allopregnanolone, which, along with related neurosteroid, modulates synaptic and extrasynaptic GABA_*A*_-receptor function (Darbra and Pallarès, 2022; Dehorter et al., 2012). By enhancing the actions of GABA, neurosteroids help orchestrate the transition from excitatory to inhibitory GABAergic signaling (Darbra and Pallarès, 2022; Dehorter et al., 2012; Mellon, 2007; Mellon and Griffin, 2002). Likewise, disruptions in neurosteroidogenesis during this critical period can alter the expression of GABA_*A*_-receptor subtypes, potentially affecting neuronal excitability and circuit formation (Mennerick et al., 2004; Mòdol et al., 2014). As development progresses, neurosteroid levels begin to decline by the third postnatal week, which in turn influences the expression of K+/Cl- cotransporter KCC2, a key mediator of the GABA polarity shift (Mòdol et al., 2014).

Exogenous neurosteroid exposure during the early postnatal development has been shown to influence the layer-specific distribution of GABAergic interneurons in the cortex, suggesting a regulatory role in the spatial organization of cortical connectivity (Grobin et al., 2003). Aberrant neurosteroid levels during this period can have lasting effects, influencing behavior during adolescence and adulthood (Gore and Gould, 2024; Gunn et al., 2013). Further, early adversity has been linked to disruptions in neurosteroid signaling, potentially altering the trajectory of neurodevelopment and increasing vulnerability to psychiatric disorders (Gore and Gould, 2024; Gunn et al., 2013; Paris and Frye, 2011). Under basal conditions, the brains response to stress originates in the paraventricular nucleus (PVN) of the hypothalamus, where corticotropin releasing hormone (CRH) is secreted from neuroendocrine cells. CRH is then shuttled to the anterior pituitary gland where along with adrenocorticotropic hormone (ACTH) is secreted by corticotrope cells in the posterior pituitary gland. ACTH stimulates the synthesis and release of glucocorticoids such as cortisol from the adrenal gland that functions as a negative feedback mechanism to terminate the stress response by inhibiting the release of CRH from the PVN. Neurosteroids also have been demonstrated to be released during stress and exert negative feedback much like glucocorticoids. Early life adversity appears to dysregulate stress-responsive circuits, impairing the ability of neurosteroids to modulate the activity of the PVN to suppress excessive stress responses in animal models (Sze and Brunton, 2020). Notably, exogenous administration of neurosteroids, such as 5α-THDOC, has shown promise in mitigating behavioral and neuroendocrine disturbances associated with early life adversity in rats, highlighting their therapeutic potential in neurodevelopmental and mood disorders (Sze and Brunton, 2020).

### Childhood/adolescence

Emerging research highlights the complex interplay between neurosteroids and developmental, psychological, and physiological processes during crucial periods of development such as the transition from childhood through adolescence. Serum allopregnanolone levels appear to be comparable between male and female infants during the first years of life (Fadalti et al., 1999). However, children diagnosed with ADHD exhibit lower levels of allopregnanolone relative to healthy peers (Sahin et al., 2022), an effect with both potential diagnostic and therapeutic relevance.

Puberty is a period marked by dramatic shifts in the levels of steroid hormones and neurosteroids. In both sexes throughout puberty there is a progressive increase in DHEA, progesterone, and allopregnanolone levels (Fadalti et al., 1999). These hormonal shifts may have critical implications for mental health; for example recent findings reveal that there is a negative correlation between DHEA levels and self-esteem (del Río et al., 2024), alongside a positive correlation between depression scores and DHEA-sulfate (del Río et al., 2024). Progesterone levels in females, meanwhile, follow a predictable trajectory during the menstrual cycle- remaining steady through the follicular phase and gradually increasing during the early to mid-luteal phases before precipitously declining in the late luteal phase (Reed and Carr, 2018).

Rodent studies have demonstrated that stress to the immune system may not only delay puberty but expedite the depletion of ovarian follicles. These effects were linked to diminished expression of the gene *Srd5a1*, which encodes for the enzyme 5α-reductase type 1, in the hypothalamus and ovaries (Bar-Sadeh et al., 2022). These findings reinforce the sensitive interaction between immune, endocrine, and neurodevelopmental systems.

### Adulthood

Neurosteroid levels are dynamically regulated across the lifespan and reproductive cycle, particularly in women. Fluctuations in the expression of δ-subunit-containing GABA_*A*_Rs have been reported across the estrous cycle and in affective disorders such as postpartum depression, major depressive disorder, and anxiety disorders (Gilfarb and Leuner, 2022; Lüscher and Möhler, 2019; Maguire et al., 2005). These alterations are frequently concurrent with changes in endogenous neurosteroid levels, suggesting that dysregulation of neurosteroid-GABAergic interactions may contribute to the pathophysiology of mood disorders (Walton et al., 2023; Walton and Maguire, 2019; Zorumski et al., 2025). Notably, women with Premenstrual Dysphoric Disorder (PMDD), demonstrate significantly higher plasma and serum levels of allopregnanolone during the luteal phase (Girdler et al., 2001), implicating potential neurosteroid dysregulation in the emergence of impaired mood regulation.

One of the most well-characterized age-related changes is the progressive decline in circulating DHEA. DHEA levels peak in early adulthood, typically during the third decade of life, and then decrease steadily with advancing age (Stárka et al., 2015). This decline contributes to the broader hormonal shifts associated with aging that occur in response to age-related depletion of organ reserve (Atamna et al., 2018), which may impact the synthesis of neurosteroid.

Over the menstrual cycle in women, neurosteroid concentrations also exhibit significant cyclical fluctuations (Kimball et al., 2019). During the early follicular phase, progesterone levels remain relatively low. As the cycle progresses into the luteal phase, progesterone concentrations rise substantially, coinciding with increasing estradiol levels as the corpus luteum becomes active. However, in the absence of fertilization, the regression of the corpus luteum leads to a sharp decline in both progesterone and estradiol to trigger menstruation.

These cyclical and age-related changes in neurosteroid levels highlight the complex regulation of the neuroendocrine environment in women. Understanding the patterns is essential for elucidating the full physiological roles of neurosteroids and their potential therapeutic implications in both reproductive and post-reproductive phases of life.

### Pregnancy and the postpartum period

Pregnancy is a state of remarkable neuroendocrine changes, during which the brain (and placenta) undergoes significant and unique adaptations to support pregnancy and ultimately coordinate a successful parturition. The brain undergoes dramatic reorganization to prepare for motherhood (Ballesteros et al., 2025; Pawluski, 2024; Pawluski et al., 2022). A hallmark of this period is the dramatic upregulation of neurosteroidogenesis, with progesterone and its potent metabolite allopregnanolone reaching peak levels as gestation progresses (Concas et al., 1998). These neurosteroids are synthesized in both the central and peripheral nervous system, with the placenta becoming the primary source of progesterone after the first trimester, following initial production by the corpus luteum until about the 9^*th*^ week of pregnancy (Cable and Grider, 2023). Subsequently it is produced by trophoblasts (Cable and Grider, 2023).

Neurosteroids are not only byproducts of hormonal flux but are themselves active neuromodulators. During pregnancy, the brain exhibits increased expression of neurosteroidogenic enzymes, a shift that enhances the synthesis of neurosteroids like allopregnanolone (Hirst et al., 2006), known for its anxiolytic and GABAergic potentiating properties. One key role of this neurosteroid surge is the suppression of the maternal hypothalamic-pituitary-adrenal (HPA) axis, effectively dampening stress responsiveness and promoting fetal development in a low-stress intrauterine environment (Brunton et al., 2009).

Importantly, neurosteroids also interface with the oxytocinergic system to finely regulate the timing of labor (Blyth et al., 2000). Oxytocin is a peptide hormone produced by the hypothalamus and released into the bloodstream by the pituitary gland. Secretion of oxytocin is initiated by neural inputs from the birth canal and brainstem to increase uterine contractions resulting in fetal dissent through the birth canal. During pregnancy, increased allopregnanolone levels exert a neuroprotective role by increasing inhibitory tone through positive allosteric modulation of GABA_A_ receptors upon magnocellular oxytocin neurons (Blyth et al., 2000). In addition to these actions, allopregnanolone also modulates the body’s endogenous opioid system (Brunton et al., 2009), providing another layer of protection against secretion of oxytocin before term. At parturition, the loss of progesterone through placental expulsion initiates the upregulation of oxytocin receptor expression in the myometrium, the smooth muscle of the uterus, facilitating coordinated uterine contractions (Grazzini et al., 1998).

The dynamic and robust changes in steroid hormone and neurosteroid levels throughout the peripartum period introduces a window of vulnerability for the emergence of mood disorders (Maguire et al., 2020; Payne and Maguire, 2019; Vesga-López et al., 2008; Walton and Maguire, 2019). The rapid decline in neurosteroid levels, particularly allopregnanolone, coincides with a sharp increase in the incidence of depressive symptoms, with postpartum women displaying significantly higher rates of depression compared to their non-pregnant counterparts (Vesga-López et al., 2008). This underscores the critical role of neurosteroids not only in maintaining pregnancy but also in safeguarding maternal mental health during and after this profound period of physiological transition. In fact, allopregnanolone analogs which act as positive allosteric modulators of GABA_A_ receptors have been FDA approved for the treatment of postpartum depression (Kanes et al., 2017; Walton and Maguire, 2019), highlighting the role of neurosteroids in regulating mental health throughout the peripartum period.

### Menopause

Menopause marks the transition during which the menstrual cycle ceases in women, signaling the end of reproductive capacity. This period is characterized by significant hormonal changes, including a decline in the levels of allopregnanolone and progesterone (Kimball et al., 2019; Slopien et al., 2018). As follicles diminish, estradiol and progesterone levels decrease, while levels of luteinizing hormone (LH) and follicle-stimulating hormone (FSH) increase in response to reduced negative feedback (Giannini et al., 2021). Additionally, dehydroepiandrosterone (DHEA) levels progressively decline with age (Giannini et al., 2021), contributing to the broader hormonal shifts observed during and after menopause.

One therapeutic strategy aimed at alleviating the neuroendocrine and psychological consequences of menopause is hormone replacement therapy (HRT) (Andréen et al., 2005). The use of neurosteroids for HRT has gained clinical attention however, outcomes related to its use have been variable. Progestogens, synthetic hormones that mimic the actions of progesterone, are frequently utilized in HRT protocols, particularly for their protective properties against endometrial cancers in women undergoing natural menopause (Weise et al., 2021). The literature pertaining to neurosteroid-based interventions for both naturally occurring and medically induced menopause is limited, with clinical studies having conflicting results (Luine, 2014; Weise et al., 2021), underscoring the need for more comprehensive and targeted research into this area.

### Older adults

Biological aging has been associated with a marked decline in the enzymatic activity required for neurosteroid synthesis. In aged rodents 5α-reductase activity is significantly reduced, impairing neurosteroidogenesis (Livingstone et al., 2014; Munetomo et al., 2015; Rosario et al., 2009; Rossetti et al., 2015). In humans, this decline is paralleled by a significant reduction in circulating neurosteroid levels, which drop substantially with age. By 80 years of age, neurosteroid concentrations are approximately 20% of those measured at 20 years of age (Orentreich et al., 1984), indicating a profound age-related reduction in neurosteroid availability.

Collectively, these findings underscore the potential contribution of impaired neurosteroidogenesis to the neurobiological changes associated with aging. However, the functional consequences of this age-related decline in neurosteroid synthesis are not yet fully understood, however, evidence suggests a potential link to both cognitive decline and mood disorders observed in older adults.

## Lifestyle modifications that alter neurosteroids

### Diet

Neurosteroids are synthesized from cholesterol (Mellon and Vaudry, 2001) (For review see Table 2) and are increasingly recognized as being modulated by dietary factors, with growing evidence supporting the role of specific nutrients in maintaining neurosteroid homeostasis. Diets rich in antioxidants (e.g., polyphenols), unsaturated fats, and probiotics have not only been shown to influence multiple cellular pathways but also function to mitigate some of the deleterious effects of environmental and emotional stressors on neurosteroid levels (Iban-Arias et al., 2022; Leri et al., 2020; Marotta et al., 2020; Scuto et al., 2024).

Metabolic dysfunction, particularly obesity (excessive body fat), is associated with altered neurosteroid profiles. Reduced levels of allopregnanolone and its precursor pregnenolone have been observed in obese individuals, with pregnenolone levels in cerebrospinal fluid (CSF), negatively correlating with body max index (BMI) yet positively correlating with cognitive measures (Ramírez et al., 2022). Dietary composition also appears to play a significant role in these outcomes. For instance, consumption of a high-fat and high-sucrose diet significantly decreased pregnenolone levels in the arcuate nucleus of the hypothalamus - a brain region central to governing energy expenditure and metabolic regulation (Ramírez et al., 2022). Moreover, a ketogenic diet, which is high in fat and low in carbohydrate content, has been reported to reduce dihydroprogesterone (DHP), allopregnanolone, dihydrotestosterone (DHT), and 3α-androstanediol (Rhodes et al., 2005).

The impact of diet on neurosteroidogenesis is particularly pronounced during pregnancy. High sugar intake prior to and during gestation alters both maternal and fetal neurosteroid profiles. For example, a high-sugar maternal diet has been shown to increase serum levels of aldosterone, 11-dehydrocorticosterone, and 11-deoxycorticosterone in maternal serum, while simultaneously increasing aldosterone in the labyrinth zone and decreasing testosterone in the junctional zone of the placenta (Seib et al., 2025). In the fetus, this diet led to elevated blood aldosterone and brain region-specific changes in neurosteroid levels, including decreased testosterone in the nucleus accumbens, suppressed corticosterone in the orbital cortex and preoptic area, and increased aldosterone in the nucleus accumbens and medial prefrontal cortex (Seib et al., 2025). Elevated aldosterone and 11-dehydrocorticosterone levels were also detected in amniotic fluid (Seib et al., 2025). Similarly, fructose consumption during pregnancy impaired neurosteroidogenesis in offspring by downregulating expression of mRNAs for *StAR, TSPO*, and *17*β-*HSD-3*, while upregulating mRNAs for *P450(11*β*)-2, 11*β-*HSD-2*, and *17*β-*HSD-1* (Ohashi et al., 2015).

Nutraceutical interventions have been proposed to offer potential to restore neurosteroidogenic balance. Palmitoylethanolamide (PEA), a saturated fatty acid naturally found in peanuts, soy lecithin, and egg yolks (Lambert et al., 2012), has been shown to exert neuroprotective effects in rodent models of post-traumatic stress disorder (PTSD) (Locci and Pinna, 2019). Administration of PEA increased brain levels of allopregnanolone, pregnenolone, progesterone, and 5α-DHP (Locci and Pinna, 2019). This was accompanied by normalization of neurosteroidogenic enzyme expression, including StAR, CYP11A1, and 5α-reductase type 1, which are typically downregulated in PTSD models (Locci and Pinna, 2019).

Collectively, these findings underscore the influence of dietary composition on neurosteroid biosynthesis and highlight the potential of dietary interventions to modulate neurosteroid profiles in both physiological and pathological states.

### Exercise

Physical activity has been recognized as a potent modulator of neurosteroidogenesis, with specific impacts shown during pain regulation, mood, and cognitive function (Scioli-Salter et al., 2016). One of the most notable neurosteroids influenced by exercise is allopregnanolone. Exercise-induced elevations in allopregnanolone have been shown to be correlated with increased pain tolerance (Scioli-Salter et al., 2016), suggesting a neuromodulatory role in stress resilience and sensory perception. Interestingly, in this study it was also demonstrated that cortisol and dehydroepiandrosterone (DHEA) levels rose from baseline following exercise; however, these increases were inversely correlated with pain tolerance, ultimately highlighting the complex and potentially divergent roles of individual neurosteroids in pain modulation (Scioli-Salter et al., 2016).

Exercise has also demonstrated benefits in aging populations, where hormonal and neurochemical shifts contribute to mood dysregulation and cognitive decline. In older adults, regular cycling was found to elevate circulating DHEA levels, an effect associated with improved mood and reduced symptoms of fatigue, tension, depression, and anger (Sonnenblick et al., 2018). Similarly, in aged rodents, a low-aerobic exercise routine led to, increased brain levels of allopregnanolone, suggesting that even mild physical activity can promote neurosteroidogenesis in the aging brain (Aoyama et al., 2019).

Further supporting the role of exercise in neurosteroidogenic balance, mild physical activity has been shown to upregulate 5α-reductase expression and levels of dihydrotestosterone (DHT) in the hippocampus - a brain region essential for learning, memory, and stress regulation (Okamoto et al., 2012). These changes may contribute to the neuroprotective and mood-stabilizing effects often observed with consistent exercise.

Together, these findings highlight the multifaceted role of exercise in regulating neurosteroid levels and support its potential as a non-pharmacological intervention for enhancing pain tolerance, mood stability, and neuroendocrine function across the lifespan.

### Meditation

Emerging evidence suggests that mindfulness-based practices can positively influence neurosteroid levels, particularly dehydroepiandrosterone sulfate (DHEAS) and its precursor DHEA, which are associated with stress resilience and mood regulation (Jørgensen et al., 2021; Nagendra et al., 2022). In a study examining the effects of a structured mindfulness-based stress reduction program, participants who engaged in 8 weeks of daily 45 min yoga and mediation session, weekly 90 min mindfulness training, and two 7 h silent retreats exhibited significantly elevated DHEAS levels 12 weeks after completing the intervention (Jørgensen et al., 2021). These findings indicate that consistent engagement in mindfulness practices may promote long-term neuroendocrine adaptations. In addition to structured programs, long-term engagement with meditation independently correlates with elevated DHEA levels. Individuals with an extensive history of regular meditation practice were found to have higher circulating DHEA concentrations compared to non-meditators, suggesting sustained neurosteroidogenic benefits associated with long-term meditation practices (Nagendra et al., 2022).

## Concluding statement

Neurosteroids are critical modulators in brain development, functioning, and resilience across the human lifespan. From early fetal development and the orchestration of postnatal synaptic pruning, through the hormonal fluctuations of adolescence, reproductive transitions, and advanced biological aging, neurosteroids dynamically influence neuroplasticity, stress responsiveness, and emotional regulation. Synthesis of neurosteroids is not only developmentally regulated but also influenced by internal physiological states and external environmental inputs, such as diet, physical activity, and psychological stress. The age-related decline in neurosteroidogenic capacity, driven in part by organ reserve depletion and diminished enzyme activity, is increasingly implicated in cognitive deterioration, mood disorders, and neurodegenerative processes.

Despite the mounting evidence highlighting their significance, neurosteroids remain an underexplored frontier in the complex field of neuroendocrinology. Understanding how neurosteroid signaling is shaped by both intrinsic biological trajectories and modifiable lifestyle factors can open new avenues for therapeutic interventions. Whether through hormone replacement, nutraceuticals, exercise, mindfulness-based strategies, or pharmaceutical treatments targeting endogenous neurosteroidogenesis, the potential to harness neurosteroids for prevention and treatment of neurological and psychiatric conditions is both promising and urgent. Future research aimed at delineating precise mechanisms, developing treatment strategies, and identifying individual differences in neurosteroid responsiveness will be essential for translating this knowledge into personalized and effective interventions.
